# 

**Published:** 1878-05

**Authors:** 


### THE AMERICAN JOURNAL OF DENTAL SCIENCE,

EDITED BY

F. J. S. GORGAS, M. D., D. D. S.,

AND

JAMES B. HODGKIN, D. D. S.

VOL. 12.—THIRD SERIES.

SURGEON GEN'L'S OFFICE
LIBRARY.
79226

BALTIMORE:
SNOWDEN & COWMAN.

LONDON:

TRUBNER & CO., 60 PATERNOSTER ROW.

1878 —1879.

### INDEX TO VOLUME XII.---THIRD SERIES.

Absurd—The Source of Artificial Teeth, - - 91
A New Blow Pipe, - - - - 184
Absorption of Iodine by the Skin, - - - 187
A Rival to Carbolic Acid, - - - - 285
Action of Chloroform on the Bladder, - - 189
Abscess of Lip from Paris Green, - - 285
Abortive Treatment of Furunculus, - - 287
American Academy of Dental Science, - 182, 451
American Dental Association, - - 86, 193, 241
Anæsthesia by Rapid Respiration, - - - 128
Antidote to Carbolic Acid, - - - 191
Antiseptics, Action of, on the Blood, - - - 186
Antiseptic and Therapeutic Properties of Boracic Acid, 240
Antiseptic Solution of Benzoic Acid, - - - 286
A New Cheap and Self-Generating Disinfectant, - 287
Artificial Respiration—Howard's Method, - 288
Astringents, Local Effects of, - - - - 135
Action of Anæsthetics, - - - - 323
A Peculiar Mouth Affection, - - - 336
Anæsthetics for Children, - - - - 376
Austen, The Late Dr. Philip H. - - - 377
Advantages and Accidents of Artificial Anæsthesia, - 379
Arsenious Acid in Dentistry, - - - 385
Address to Graduates of Balto. College of Dental Surgery, 395
Alveolo-Dental Abscess, - - - - 404
Amalgam for Filling, - - - - 418, 424
A New Elastic Gum, - - - - 422
Amalgam, - - - - - - 424
An Early Symptom of Tabes Dorsalis, - - 428
A New Disinfectant, - - - - - 429
Absorption of Lime Salts, - - - - 431
Alcohol Question in England, - - - 461
Alumni Meeting of Balto. College of Dental Surgery, 468
Alumni Meeting of Ohio College of Dental Surgery, 469
Anæsthetic for Children, - - - - 477
Address before the Alumni Association of Baltimore College of Dental Surgery, - - - - - 23
Anæsthetics, Inquiries Concerning, - - - 39

Alumni Association Meetings, - - - 468
Alumni Association of Balto. College of Dental Surgery, 525
Address, Dr. J. Hall Moore's, - - - - 493
Beach, Henry E., D. D S., - - - - 31
Brinton, John H., M. D., - - - - - 132
Black, G. V., D. D. S., - - - - 456, 481
Baltimore College of Dental Surgery, - - 468, 552
Butler, C. S., D. D. S., - - - - 168
Brain Weight, Relation of, to Mental Ability, - - 48
Blepharoplasty, - - - - - 466
Boracic Acid, Antiseptic and Therapeutic Properties of, 240
Bael as an Astringent, - - - - 336
Bleaching Teeth, - - - - - 364
Borax as an Antiseptic, - - - - 479
BIBLIOGRAPHICAL.
House Air—The Cause and Promotion of Disease, - 45
Proceedings of Southern & American Dental Conventions, - - - - - 143
Questions on the Structure and Development of Human Teeth, - - - - - 236
Physicians Visiting List for 1879, - - 334
Urethral Stricture, - - - - 334
The Intra-Venous Injection of Milk, - - 334
Treatment of Genito-Urinary Organs, - - 380
The Scientific American, - - - - 381
The American Agriculturist, - - - 382
Manual of Medical Electricity, - - - 383
Virginia Medical Monthly, - - - 383, 474
New Electrodes and Battery, - - 383
Hunter's Mechanical Dentistry, - - - 472
Advantages and Accidents of Artificial Anæsthesia, 473
Facial Neuralgia, - - - - - 473
Elements of Dental Materia Medica and Therapeutics, 475
Treatment of Placentia Prævia, - - 475
Magitot's Treatise of Dental Caries, - - 475
Chase, Henry S., D. D. S., M. D., - - - 18, 320
Cockerille, S. J., D. D. S., - - - - 23
Carbonic Acid Gas Baths. - - - - 45
Cerebral Localization, - - - - - 47
Children's Teeth, Care of - - - - - 77
Chloride of Calcium, - - - - - 85
California Dental Association, - - - 85
Curious Development of Teeth, - - - - 92
Cell and Protoplasm, - - - - 96
Cheiloplasty, - - - - - 132
Chloroform and the Dentists, - - - 140
College Changes, - - - - - 143
Conventions, Associations, Recreation, - - 185

Chloroform Administration, Rules for - - 185
Chloroform Narcosis Cured by Nitrite of Amyl, - 192
Conservative Treatment of the Pulp, - - - 216
Chloral Hydrate in Tetanus, - - - - 283
Combined Use of Chloroform and Morphia, - - 286
Chloroform Narcosis, Prevention of, - - - 286
Cutter, Ephraim, M. D., - - - - - 311, 470
Chupein, Theo. F., D. D. S., - - - - 327
Chloroform Administration, - - - - 335
Chloroform and Nitrite of Amyl, - - - 336
Chloramyl—A New Anæsthetic, - - - - 371
Case of Third Dentition, - - - - 384
Chlorate of Potash Poisoning, - - - - 430
Close of Volume, - - - - - 569
Conservative Surgery, - - - - 432
College Commencements, - - - - 468, 522
Class Address, - - - - - 561
Compound Hare-Lip, - - - - - - 567
Camphor as a Hypnotic, - - - - 478
Chloral as a Revulsive, - - - - - 480
Cuprum Ammoniatum in Neuralgia, - - - 480
Convulsions from a Hair, - - - - - 480
Case of Oral Surgery, - - - - - 519
Commencement (39th) of Baltimore College of Dental
Surgery, - - - - - - 522
Causes of Decay of the Teeth of Americans, - - 71
Congenital Cleft Palate, - - - - 289
Diffusion of Carcinoma by the Nerves, - - - 427
Deaths from Anæsthetics, - - - - - 429
Disadvantages of Salicylic Acid as a Mouth-Wash, - 432
Dental Neuralgia, - - - - 456, 481
Decision in a Vulcanite Suit, - - - - 479
Dental Operations, The Use of Chloroform in, - 542
Dentistry—Is it a Specialty of Medicine? - - 500
Dental Profession—Is it Crowded? - - - 28
Diagnostic Value of the Soft Palate in Pathological
Conditions, - - - - - - - 161
Development of Tœnia Solium in Man, - - 47
Decay of the Teeth of Americans, Causes of, - - 71
Dental Association Meetings, - - 85, 86, 87, 143
Dangers from Salicylic Acid, - - - - 93
Dental Decay and Filling Materials Considered in their
Electrical Relations, - - - - - 105
Dangers of Horseback Exercise, - - - - 186
Disadvantage of Salicylic Acid as a Dentifrice, - 192
Death Under Ether, - - - - - - 239
Dentition as one of the Causes of Cholera Infantum, - 316
Dental Amalgams, Some Experience with, - - - 320

English Dentistry and the Chemist, - - - - 182
Effects of Symphon Cicatrization, - - - - 91
Elimination of Mercury, - - - - - 92
Eames, W. H., D. D. S., - - - - - - 230
Effects of Dental Operations During Period of Utero Gestation, 358
Eyes, The Care of the, - - - - - - 464
Effect of Diet on Liquor Drinking, - - - - 477
Effects of Ligation of the Thighs in Obstinate Epistaxis, 528
Epithelioma, - - - - 466
Fuchs, Alfred, D. D. S., - - - - 374
Flagg, J. Foster, D. D. S., M. D., - - - 1, 347
Fundenberg, Walter H., D. D. S., - - - - 216
Filling Proximal Cavities in Bicuspids and Molars, - 413
Female Physicians, - - - - - 430
Food and the Human Body, - - - 95
Foul Breath, - - - - - - 529
Gold, Lectures on, - - - - 35, 272
Gases, Liquefaction of, - - - - - 63
Goodwillie, D. H., M. D., D. D. S., - - - 49
Gelseminum Sempervirens in Neuralgia, - - 90
Ginley, Dr. A. H., - - - - - 128
Gold Can be so Manipulated as to Make Operations Permanent, - - - - - - 268
Georgia State Dental Society, - - - 279
Gunning, Thos. Brian, D. D. S., M. D., - - - 289
Grammer, Rev. Julius E., D. D., - - - 395
Grant, H. M., D. D. S., M. D., - - - - 519
Hodgkin, Prof. Jas. B., D. D. S., - 28, 35, 71, 97, 111, 122, 173, 179, 272
Holmes, Dr. A. M., - - - - - 328
Harlan, Dr. A. W., - - - - - - 82
Hurd, E. P., M. D., - - - - - 134
Hope for the Dental Profession,—The Cummings' Patent, 137
Hodson, J. F. P., D. D. S., - - - - - 145
Howard, F. E., D. D. S., - - - - - 157
Hypodermic Injections of Chloroform, - - 192
Howard's Method of Artificial Respiration, - - 288
Hard Rubber Appliances for Congenital Cleft Palate, 289
How to Make Nerve Bristles, - - - 327
Healing of Pieces Separated from the Human Body, 432
Heavy Sentence of a Dentist, - - - 432
Harris, E. N., D. D. S., - - - - 451
Is the Dental Profession Crowded? - - - - 28
Iodoform as a Local Application, - - - 188
Ingersoll, Dr. L. C., - - - - - 210, 256
Impurities and Tests of Chloroform, - - - - 237
Inflammation, Abscess, and Necrosis—Cause and Treatment, 433
Imbedding of a Piece of Tooth in the Tongue, - 476

Is Dentistry a Specialty of Medicine? - - 500
Influence of Cheeks, Tongue and Lips on Gums and Jaws, 116
Klump, G., D. D. S., - - - - 77
Lancing the Gums, - - - - - - 422
Liquefaction of Gases, - - - - 63
Lead Poisoning from a Curious Source, - - 48
Lesions of the Trifacial from Dental Diseases, - 49
Local Effects of Astringents upon the Blood Vessels, 135
Love, W. A., M. D., - - - - - 161
Lead Poisoning by Flour, - - - 191
Loss of Blood in Surgical Operations, - - 374
McElroy, Z. C., M. D., - - - - 329
Mal-Nutrition,—Its Causes, - - - - 235
Metric System, - - - - - 111
Mechanical Dentistry, - - - - - 168
Malformation of the Skull in Epileptics, - - 190
Maryland and District of Columbia Dental Association, 298, 330, 337
Nævus, or Effects of Dental Operations during Utero-Gestation, - - - - - - 358
Newest of our Creeds. - - - - - - 87
New Method of Plugging. - - - - 189
Neuralgia, Nature and Treatment of, - - - 283
Nowlin, J. H., M. D., - - - - - 316
Nitrite of Amyl and Chloroform as an Anæsthetic, - 336
Neuralgic Tooth-Ache, - - - - 480
New Method of Attaching Gold Crowns to Natural Roots of Teeth, - - - - - - 425
OBITUARY NOTICES.
Philip H. Austen, M. D., D. D. S., 332, 377, 576
Charles Knower, D. D. S., M. D., - - 333
John H. McQuillen, M. D., D. D. S., - - 571
Odell, Frank, D. D. S., M. D., - - - 126
Observations and Experiments Connected with Oral Electricity, - - - - - - 18
Opium vs. Coffee, - - - - - 186
Offerings, - - - - - - - 145
Oxygen as a Cure for Hydrophobia, - - 190
Plastic Filling, and the Basal Principles of the New Departure, - - - - - 1
Plastic Filling as a Power in Dentistry, - - - 347
Professional Hobbyism, - - - - 31
Proximate Causes of Decay of the Teeth of Americans, 97
Pennsylvania State Dental Society, - - - 130
Physiology of the Lymphatic System, - - 329
Pyorrhea Alveolaris,—A Case in Practice, - - 230
Poisoning by Chlorate of Potash, - - - 430
Paper Teeth, - - - - - - - 478

| | |
|---|---|
| Palmer, S. B., D. D. S., | 105 |
| Prothro, Dr. S. M., | 358 |
| Porter, J. M., D. D. S., | 433 |
| Pierce's Golden Medical Discovery, | 47 |
| Qualification of the Dental Student, | 280 |
| Relation of Brain Weight to Mental Ability, | 92 |
| Replantation of Teeth, | 93 |
| Restoration of Inferior Maxilla after Removal, | 122 |
| Requisite Knowledge for the Practice of Dentistry, | 126 |
| Rosenstein, Dr. A., | 135 |
| Restoration after Necrosis and Removal of Inferior Maxilla, | 179 |
| Rules for Chloroform Administration, | 185 |
| Relations of Dental Pulp to other Tooth Tissues, | 210, 256 |
| Results of Some Recent Experiments, | 277 |
| Rubber Suit, | 282 |
| Royce, W. E., D. D. S., | 446 |
| Relations to Life of Aerostatic Pressure, | 46 |
| Substance of Lectures on Gold, | 35, 173, 272 |
| Specialism, | 42 |
| Southern Light, | 44 |
| South Carolina Dental Association, | 86 |
| Southern Dental Association, | 87, 227 |
| Salicylic Acid as a Dentifrice, | 95 |
| Statistics of Insanity, | 93 |
| Sauer, Dr. C., | 116 |
| Stomatitis Materna, | 134 |
| Strengthening Rubber Dentures and Repairing Broken Plates, | 157 |
| Stilbeine for Cleaning Instruments, | 238 |
| Skin Grafting in the Colored Races, | 239 |
| Smith, D. D., M. D., D. D. S., | 404 |
| Skulls of Women, | 431 |
| Sugar, | 476 |
| Smith, C. Stoddard., D. D. S., | 500 |
| Sulphate of Quinine, | 284 |
| Salicylic Acid and Borax, | 285 |
| Sanford, Geo. E., M. D., | 371 |
| Taft, J., D. D. S., | 364 |
| Treatment of Deciduous Teeth. | 446 |
| Third Dentition—A Case of, | 384 |
| Treatment of Stomatitis Materna, | 134 |
| The Intra-Venous Injection of Milk, | 334 |
| Turpentine Vapor in Accidents from Chloroform, | 384 |
| The Uses of Pain, | 467 |
| The Care of the Eyes, | 464 |
| The Safety Pocket, | 47 |
| The Dental and Oral Science Magazine, | 89 |
| Treatment of Earache, | 94 |

Tubercular Ulcer of the Tongue, - - - - 96
Thymol, - - - - - - - - - 177
Thymol as an Antiseptic, - - - - - 188
To Utilize Gutta-Percha Scraps, - - - 189
The Physiological Action of Aconite, - - 189
The Metric System in a Nut-Shell, - - - 223
Treatment of Furruncles by Arnica, - - 238
Therapeutic Value of Nitrite of Lead, - - - 238
The New Rubber Suit, - - - - 282
Treatment of Wounds, - - - - - 284
To Blister Quickly, - - - - - 428
Use of Dissimilar Metals in Filling a Tooth, - - 82
Uranine—A New Aniline Color, - - - 425
Vegetable Parasites, - - - - - - 191
Virginia State Dental Association, - - - 275, 511
Vermillion, - - - - - - - 240
Warning Against Hypodermic Use of Morphia, - 430
Webb, M. H., D. D. S., - - - - - - 268
Wigglesworth, Edward., M. D., - - - 223
Write It, - - - - - - - 44

### To Readers and Correspondents.

Communications intended for publication, and exchange journals and papers, should be addressed to the Editor of the American Journal of Dental Science, 259 N. Eutaw St., Baltimore, Md. All letters relating to the business of the journal, or containing cash remittances, to be directed to Messrs. Snowden & Cowman. Articles for publication should be received by the editor at least three weeks previous to the first day of the month on which the number in which it is designed for them to appear, is issued.

### CONTENTS:

Specialism .......... 42

Write It .......... 44

Southern Light .......... 44

### BIBLIOGRAPHICAL.

House Air—The Cause and Promotor of Disease .......... 45

### MONTHLY SUMMARY.

Carbonic Acid Gas Baths .......... 45

Relations to Life of Aerostatic Pressure .......... 46

Development of Tænia Solium in Man .......... 47

Pierce's Golden Medical Discovery .......... 47

Cerebral Localization .......... 47

The Safety Pocket .......... 47

Lead Poisoning from a Curious Source .......... 48

Relation of Brain Weight to Mental Ability .......... 48

☞ The American Journal of Dental Science will contain fifty pages of reading matter in each number, making a volume of about

*Six Hundred* pages

EXCLUSIVE OF ADVERTISEMENTS

THE

### AMERICAN JOURNAL

OF

### DENTAL SCIENCE.

The *Journal* is issued on the first of each month and contains not less than *fifty* pages of reading matter in each number, exclusive of advertisements, making a volume of *six hundred* pages per annum.

In each number will be found Original Communications, Correspondence, Selected Articles, Monthly Summary, Bibliographical Notices and Editorial Matter, and every effort will be exerted to render the *Journal* worthy of the patronage of the Dental profession.

A generous co-operation is respectfully solicited from the members of the profession; and as the *Journal* has now a printing office of its own, which materially lessens the cost of its publication, it will be issued at the reduced subscription price of

**$2.50 Per Annum in Advance.**

Communications are respectfully solicited on all subjects pertaining to the practice of dentistry, as well as reports of cases occurring in dental practice.

### RATES OF ADVERTISING.

| | 1 MO. | 2 MO. | 3 MO. | 6 MO. | 12 MO. |
|---|---|---|---|---|---|
| 1 PAGE. | $15 00 | $22 50 | $30 00 | $50 00 | $100 00 |
| ½ " | 10 00 | 15 00 | 20 00 | 30 00 | 50 00 |
| ¼ " | 7 00 | 11 00 | 15 00 | 20 00 | 30 00 |
| ⅛ " | 5 00 | 8 00 | 11 00 | 15 00 | 20 00 |

All original communications designed for publication, works for review, new instruments for notice, exchanges, &c., should be directed to the editor, 259 N. Eutaw St., Baltimore, Md.

All advertisements and subscriptions should be addressed to

SNOWDEN & COWMAN,

*Publishers of the Am. Journal of Dental Science.*

86 W. FAYETTE ST. BET. CHARLES & LIBERTY STS.,

BALTIMORE, MD

